# Chinook salmon (*Oncorhynchus tshawytscha*) genome and transcriptome

**DOI:** 10.1371/journal.pone.0195461

**Published:** 2018-04-05

**Authors:** Kris A. Christensen, Jong S. Leong, Dionne Sakhrani, Carlo A. Biagi, David R. Minkley, Ruth E. Withler, Eric B. Rondeau, Ben F. Koop, Robert H. Devlin

**Affiliations:** 1 Fisheries and Oceans Canada, West Vancouver, BC, Canada; 2 University of Victoria, Victoria, BC, Canada; 3 Pacific Biological Station, Fisheries and Oceans Canada, Nanaimo, BC, Canada; Northwest Fisheries Science Center, UNITED STATES

## Abstract

When unifying genomic resources among studies and comparing data between species, there is often no better resource than a genome sequence. Having a reference genome for the Chinook salmon (*Oncorhynchus tshawytscha*) will enable the extensive genomic resources available for Pacific salmon, Atlantic salmon, and rainbow trout to be leveraged when asking questions related to the Chinook salmon. The Chinook salmon’s wide distribution, long cultural impact, evolutionary history, substantial hatchery production, and recent wild-population decline make it an important research species. In this study, we sequenced and assembled the genome of a Chilliwack River Hatchery female Chinook salmon (gynogenetic and homozygous at all loci). With a reference genome sequence, new questions can be asked about the nature of this species, and its role in a rapidly changing world.

## Introduction

For many, the Pacific salmon are cultural icons and represent a heritage bridging the Pacific Ocean and the lands they inhabit. The Chinook salmon (*Oncorhynchus tshawytscha*) is the largest of the Pacific salmon [1] and is a primary target in many recreational and commercial fisheries. They are prized for their size, resilience, and the quality of their flesh. Their wide distribution, relatively recent population decline, and ability to survive adversity make the Chinook salmon of great interest to many people. Chinook salmon are also a keystone species influencing forest and stream ecosystems [2].

Chinook vary extensively in life-history characteristics and subsequently occupy a wide range of habitats. Historically, the Chinook salmon habitat included a North American distribution from Point Hope, Alaska in the North, to the Ventura River (California) in the South [3]. In Asia, the northern habitat limit was the Anadyr River and the southern limit was Hokkaido in Japan [1]. From the sparse estimates of historical abundance (before European colonization), it is thought that as many as 6 million Chinook flooded the western, North American tributaries of the Columbia River, while 2 million returned to Sacramento River tributaries in California [3].

In 2001, around 5 percent (~325,000) of the historical and wild Chinook salmon run returned to the Columbia River [3]. The Chinook salmon is the least abundant Pacific salmon species and is currently of conservation concern in southern British Columbia and much of Washington State. Populations have been listed under the American Endangered Species Act and are under review by the Canadian Committee on the Status of Endangered Wildlife in Canada. In the Salish Sea, Chinook abundance has decreased by 60 percent between 1984 and 2010 [4]. From commercial catch records that incorporate wild and hatchery origin fish, Chinook salmon abundance, in general, appears to be on the decline from 1925 to 2009 in the eastern North Pacific Ocean (with a caveat of intentional reduced catch after 1989) [5].

The wild Chinook salmon declines are possibly related to freshwater habitat degradation from mining and logging operations, the construction of dams blocking upstream habitat, oceanic regime shifts, extensive hatchery production, mixed-stock harvest, and climate change. Hatcheries have long been used to mitigate these declines in Chinook and other Pacific salmon. In Washington state, there are now (as of 2017) 83 state, 45 tribal, and 12 federal hatcheries [6]. In 2017, 101,599,671 Chinook smolts were released into the Columbia River Basin from hatcheries [7].

Chinook Salmon are nektonic (not dependent on ocean currents) feeders that occupy the highest trophic level among the Pacific salmon [8]. This is consistent with their heavy dependence on freshwater rearing environments and tendency for coastal migrations in the marine environment, where they feed on squids and fishes. Their coastal distribution increases vulnerability to the anomalous environmental and biotic conditions associated with current and wind-mediated weather events such as the development of the warm oceanic ‘blob’ in the NE Pacific Ocean in 2014 [9]. Similarly, the anthropomorphic and climatic alterations of coastal waters influence the predators as well as the prey of Chinook Salmon. Chasco [10] demonstrated that increased marine mammal (killer whale, seal, sea lion) predation in the forty years between 1975 and 2015 likely more than accounted for all harvest reductions in fisheries over that time period. Thus, the successful recovery and/or expansion of predator populations may have precluded success in conservation efforts conducted for Chinook Salmon. Whereas dam construction and freshwater habitat destruction combined with hatchery supplementation and high harvest levels may have caused much of the Chinook Salmon decline observed prior to 2000 [11], other factors associated with coastal climate and ecosystem changes may be currently important.

The extensive phenotypic variation exhibited by salmonids coupled with their demonstrated ability to colonize new habitat makes them of interest in the study of adaptive processes [12–14]. A genome duplication (which occurred in an ancestral species around 90 million years ago) may underlie both the Chinook salmon’s abilities to adapt to environmental variation on geographically fine scales, and the broad, environmentally-mediated phenotype plasticity seen in Chinook salmon [15,16].

The ~90 million year old genome duplication still reverberates in modern salmonid genomes because of ongoing multivalent pairing during meiosis and recombination between some duplicated (homeologous) chromosomes. This process maintains sequence similarity and confounds DNA sequencing and assembly in some genomic regions [17]. In other regions, the ongoing diploidization of duplicated loci has created an evolutionary canvas for specialization and redundancy that likely underlies the adaptivity of modern salmonids. Thus, the reward for successfully defining the poorly differentiated homeologous regions to provide a complete understanding of salmonid genomes may be an unlocking of the secrets of salmonid adaptation and survival.

Understanding the Chinook salmon greater may benefit humanity culturally, economically, and help preserve a heritage built before written language. A Chinook salmon reference genome would offer researchers clarity when interpreting their results and when integrating information from other sources and researchers. It would also allow them to focus on research (e.g. detailed gene expression in response to environmental conditions, broad-scale population genetic responses, etc.) that might otherwise be limited in scale and difficult or impossible to undertake.

More specifically, a genome contextualizes genes in terms of order and orientation, proximity to regulatory regions, and copy number. This contextualization is necessary for genome-wide-association studies, identifying major chromosomal changes like inversions and translocations, and identifying regions of the genome influenced by population demographics or evolutionary processes. It also allows for comparative analyses between species with completed genome sequences.

These types of analyses are important for conservation [18,19] and aquaculture efforts [20–22]. For conservation, identifying regions of the genome that differentiate populations from one another, may help define populations better and allow fishery managers to make more refined decisions. From an aquaculture perspective, identifying underlying genetic variation responsible for production traits, via genome-wide-association studies, would be desirable because marker assisted selection could then be used to the farmer’s benefit [22]. Whether asking a simple question, such as if Chinook salmon have a particular gene (or how many copies of that gene), or asking a tough question, such as how the environment interacts with the genome through epigenetics, a genome makes it possible and easier to answer.

In this study, we sequenced and assembled the genome of a Chilliwack River Hatchery female Chinook salmon and generated an extensive multi-tissue transcriptome (from the same individual that was used to generate the genome). In addition, we performed several analysis with the new genome sequence, including an analysis to measure completeness, duplicated regions of the genome, repetitive elements, and a comparison to the coho salmon genome.

## Materials and methods

### Samples

A Chinook salmon mitotic gynogen (an individual that is completely homozygous for every genomic loci) was produced at Fisheries and Oceans Canada in West Vancouver using salmon from the Chilliwack River Hatchery (please see Table 1 for details, NCBI BioSample: SAMN07843558). A mitotic gynogen was used because genome assemblies are often improved when allelic variation is removed [23]. Mitotic gynogenesis was undertaken following procedures described by [24]. Briefly, eggs were fertilized with UV-irradiated sperm and pressure shocked (10,000 psi) in batches at 30 min intervals between 5 and 7 hours postfertilization. Surviving progeny were fin clipped and genotyped using a panel of 16 microsatellites to identify those that were homozygous at all loci and possessed no paternal genotypes. A gynogen (DE9421) was grown (see below for conditions) to a size of 58 g, at which time 18 tissues (head kidney, adipose, red muscle skin, ovary, heart, white muscle, pyloric caeca, mid gut, stomach, spleen, liver, hind gut, left eye, pituitary, whole brain, upper jaw, lower jaw, gill) were collected and stored frozen at -80°C in RNAlater. DNA was isolated using a phenol/chloroform extraction as per Thermo Fisher Scientific’s protocol for genomic DNA preparation from RNAlater preserved tissues, and RNA was purified using the Qiagen RNeasy kit. The DNA and RNA was isolated from the same individual.

**Table 1:** Sample information.

NCBI BioSample	Geographic Location	Date of Sampling	Phenotypic Information	Key Features (physiological/ biochemical)
SAMN07843558	Chilliwack Hatchery, BC	December 7, 2016	Weight: 58.32 g Length: 17.5 cm	Mitotic Gynogen

All animals were reared in compliance with Canadian Council on Animal Care Guidelines, under permit from the Fisheries and Oceans Canada Pacific Region Animal Care Committee (under Ex.7.1). Chinook salmon were grown in aerated fresh well water in 270–3000 L tanks and fed hourly as fry and to satiation 3 times daily as parr with stage-appropriate manufactured salmon feed (Skretting Canada Ltd.). At a size of approximately 10 g, fish were withheld from food for 24 hours before being anaesthetized in 100 mg/L tricaine methanesulfonate (TMS) buffered with 200 mg/L sodium bicarbonate, then tagged with a passive integrated transponder tag and adipose-fin clipped. Fish were allowed to recover for 24 hours before refeeding. Following genotyping to identify homozygous gynogens, animals were grown to a size (see above) where multiple tissues could be cleanly dissected; at which time a single selected fish was euthanized with TMS and sodium bicarbonate, then rapidly (< three min, Pacific Region Animal Care Committee management procedure 3.7) team dissected to harvest 18 tissues for DNA and RNA extraction as above.

### Sequencing and quality control

High molecular weight DNA (see above) was sent to the McGill University and Génome Québec Innovation Centre. At the centre, a genomic DNA shotgun library was prepared using a KAPA Hyper Prep Kit (KAPA Biosystems). The library was then sequenced at the centre on two lanes of an Illumina HiSeq 2500 machine using the HiSeq Rapid mode (PE 250 bp). RNA (see above) was sent to the McGill University and Génome Québec Innovation Centre where (NEB) mRNA stranded libraries were constructed. Three tissues were barcoded, combined, and sequenced per Illumina HiSeq4000 (PE 100bp) sequencing lane with six lanes being used in total.

Three additional Illumina Nextera mate-pair genomic DNA libraries were prepared at the McGill University and Génome Québec Innovation Centre. Fragment sizes of 3 kb, 5 kb, and 10 kb were attempted for each mate-pair library respectively. Each library was sequenced on a single HiSeq 2500 lane (PE 125 bp). PacBio libraries (9 genomic DNA libraries) were also prepared at the McGill University and Génome Québec Innovation Centre using SMRTbell Template Prep Kit 1.0 (size fractionation of 15–20 kbp). These libraries were sequenced on 52 SMRT cells and 7 Sequel SMRT cells.

The quality of the sequences generated from the PE 250 bp and all mate-pair libraries was assessed using FastQC [25]. Trimmomatic [26] was used to trim the sequences, remove low quality reads, and remove adapter contamination. The following parameters were specified for the mate-pair libraries PE, ILLUMINACLIP:TruSeq3-PE-2.fa:2:30:10, ILLUMINACLIP:NexteraPE-PE.fa:2:30:10, LEADING:28, TRAILING:28, SLIDINGWINDOW:4:15, and MINLEN:75. The PE 250 bp library had the same parameters except the Nextera adapters were not checked and the minimum length was set to 200 bp.

### Genome assembly

ALLPATHS-LG [27] version 52488 was used to generate a preliminary assembly from the Trimmed PE 250 bp and mate-pair library sequences. Default settings were used for CacheLibs.pl and CacheGroups.pl (ALLPATHS-LG scripts that prepare the data for the assembly). For the CacheToAllPathsInputs.pl script: five libraries (two PE 250 bp libraries based on the sequencing lane, and three mate-pair libraries) were set for the GROUPS option, 20x coverage was set for each of the PE 250 bp libraries and 11x coverage was set for each of the mate-pair libraries using the COVERAGES option, the GENOME_SIZE option was set to 2,300,000,000, and the PLOIDY was set to one since the genome originated from a gynogenetic female. Estimates of the fragment size and insert size, for the different libraries, were generated by aligning 1000 sequences from each library to the coho salmon genome (GenBank assembly accession: GCA_002021735.1) and using custom scripts. All custom perl and python scripts can be found in S1 File.

Once the preliminary files were generated, RunAllPathsLG was used to generate the initial assembly (CLOSE_UNIPATH_GAPS option set to False). Several trimming lengths, filtering options, and coverages were explored, but the reported parameters were chosen since they produced the longest contigs and scaffolds. This assembly was then used as the reference for PBJelly [28] version 15.8.24, a program used to incorporate PacBio sequences.

PacBio sequences were removed from the dataset if they were shorter than 5,000 bp. The blasr [29] settings in PBJelly were set to: -misMatch 8, -sdpTupleSize 8, -minPctIdentity 75, -bestn 1, -nCandidates 10, -nproc 8, -maxScore, -500, -noSplitSubreads. All parameters for PBJelly were the default settings. The improved assembly, produced by PBJelly, was then used by another program that also incorporates PacBio sequences. First, Canu [30] version 1.4 was used to correct the PacBio reads with a minimum length set to 2000 bp (minReadLength = 2000). The corrected PacBio reads and the improved assembly were then used by the SSPACE version 1.1 program [31] with default settings to further increase the scaffold length.

After using SSPACE to further incorporate PacBio data, the scaffolds were placed onto chromosomes based on two genetic maps [32,33] and synteny between the scaffolds to two NCBI assembled genomes (Atlantic salmon: GenBank GCA_000233375.4, [34], and rainbow trout: GenBank GCA_002163495.1); when information from the genetic map was in agreement with the synteny information. First, sequence data was extracted from both genetic maps using custom scripts and the genetic marker sequences were aligned to the Chinook scaffolds, Atlantic salmon genome, and rainbow trout genome using both BWA mem [35] (default settings) and Megablast in blastn version 2.2.31+ [36,37] (-outfmt 6, -max_hsps 2, -max_target_ses 4, -evalue 0.01). These alignments were filtered based on quality scores (for bwa alignments, mapq > = 1; for blast, a minimum percent identity of 93 and minimum alignment length of 70 was used for the [32] map, and 95 and 93 for the [33] map—because the markers differed in length), and based on the number of best alignments (only one was allowed for the alignments to the sequenced genomes).

The Chinook salmon scaffolds were also aligned to both the Atlantic salmon and rainbow trout genomes using two alignment programs; nucmer version 3.1 in Mummer [38] (default settings), and Megablast (-evalue 0.001, -max_hsps 4000, -num_alignments 5, -word_size 40, -perc_identity 94) when some scaffolds appeared to not align when just using nucmer. The alignments between the scaffolds and the genomes were then filtered based on minimum length (250 bp), minimum percent identity (92% identity for nucmer and 94% identity for Megablast), and linearity (the starting position of a scaffold needed to concordantly increase or decrease in nucleotide position relative to the genomic position for a single alignment or multiple alignments for at least 3500 bp (minal), 12.5% the total length of the scaffold (minl), could not jump more than 10% of the total length of a scaffold (smax), and could not jump more than 1% of the chromosome (cmax) using custom scripts).

For each linear alignment (including those with multiple smaller alignments), the approximate coordinates of where the chromosome aligned to the scaffold and also where the scaffold aligned to the chromosome was determined programmatically based on starting and ending positions of the entire region. The number of scaffolds or scaffold regions was reduced by only returning the best, in terms of alignment length, for a particular region of a chromosome. If the scaffold mapped to multiple locations either on the same or different chromosomes, they were manually inspected (please refer to S1 File for more details). In these cases, the region of alignment was manually determined, and the chimeric scaffold was broken.

A script was used to integrate genetic map information with the scaffold and genome alignments (referred to as synteny information below). For each syntenic region, marker, linkage group, and centimorgan information was added if available. The syntenic regions were then manually inspected for order based on the genetic map, and only accordant scaffolds were used to generate the order of these scaffolds on each chromosome. This procedure was performed with both the Atlantic salmon genome and rainbow trout genome separately. The two orders were compared programmatically and then manually to coalesce the two versions using alignments produced by nucmer. The Megablast alignment version was used to add scaffolds that were missed with the nucmer versions. A script was then used to generate the chromosome sequences from the scaffold order and the scaffold sequences. This is referred to as the Chinook genome below.

### Genome comparison and features

The Chinook genome was then aligned to the coho salmon (GCA_002021735.1) genome using Megablast (-evalue 0.0001, -max_target_seqs 3, -max_hsps 20000, -outfmt 6, -word_size 40 -perc_identity 90) and filtering nonlinear alignments using a custom script (described above with the following parameters: smax 0.01, cmax 0.01, minl 0.01, minal 500000). The comparison with the coho salmon was chosen (with the permission of the authors) because it is the most closely related species to the Chinook salmon. The alignments were then visualized in R [39] using the ggplot2 package [40]. The Chinook genome was also aligned to the genetic map used to generate it using Megablast (-outfmt 6, -max_hsps 2, -max_target_seqs 4, -evalue 0.01). These alignments were filtered based on a minimum percent identity of 94 percent, minimum alignment length of 92, and a minimum difference in quality score between the best and second best alignment of one. The filtered alignments were then used to identify the locations of the centromere on each chromosome based on the genetic map produced by [33].

To identify duplicated regions (homeologous) of the Chinook genome, SyMAP [41] was used to align a masked version of the genome (see section 2.5) to itself and identify duplicated blocks using the following parameters: merge_blocks = 1, nucmer_only = 1, and mindots = 20. The alignments produced by SyMAP were filtered based on linearity (smax 0.01, cmax 0.01, minl 0, minal 2000). The filtered alignments were then used to find the orientation of each block and find the average percent identity for million bp windows along the genome. The average percent identity was found by first finding the total alignment length (a count of all non-overlapping nucleotides that aligned in a window) for a window, and then weighting the percent identity of an alignment by the fraction of the total length that each alignment contributed.

### Repetitive DNA elements

Regions with high percentages of repetitive sequence were identified by first generating a masked Chinook salmon genome, and then by identifying the number of nucleotides that were masked in million bp windows. In order to mask the genome, first a repeat library was generated guided by the methodology of [34]. The methodology is described in the following paragraphs.

From the Atlantic salmon repeat library [34], 2,005 repetitive sequences were taken and combined with 548 repetitive sequences from the RepBase database [42]. The RepBase sequences originated from the Salmoniformes family, and excluded simple repeats (downloaded January 13, 2017). RepeatModeler v1.0.8 [43] was also used together with the ALLPATHS-LG initial assembly in a de novo approach, which identified 1,124 repetitive sequences.

The repetitive sequences were then aligned to the Chinook genome with BLASTN v2.2.28+ [36]. A wordsize of 7 was used and the dust filter was turned off in an effort to detect older, longer repeat copies. If three or more high-scoring segment pairs (HSPs), of at least 80% of the length of a given preliminary repeat library sequence, were found on at least three separate contigs, the sequence was classified as high-confidence (HC). If more than nine 100 bp HSPs were identified on separate contigs, the query repeat sequence was classified as low-confidence (LC). All other sequences were removed. In order to isolate repetitive sections of LC sequences, the sequences were split wherever the number of long (80 bp or longer) HSPs overlapping a given LC sequence base dropped below 10 for 10 consecutive bases. Low-coverage sequences were then removed from the split LC sequences.

Superfluous sequences were removed from the repeat library using a redundancy-removal procedure. All of the sequences, after the above filtering, were compared to each other using an all-by-all BLASTN search. For any alignment between two HC sequences or between LC sequences, the shorter of the two sequences was removed if there existed a set of 80 bp or longer HSPs that: i) all possessed a percent similarity of at least 80%, ii) overlapped each other by no more than 15bp, and iii) covered more than 80% of the length of the shorter sequence. The same procedure was performed on alignments between HC and LC sequences after the initial filtering of superfluous sequences, but only LC sequences were removed.

Annotation of the repeat library consisted of the identification and removal of non-Transposable Element (non-TE) host genes followed by the classification of TEs into the taxa proposed by [44]. First, BLASTX was used to align repeat library sequences to the protein sequences from both the REPET-formatted RepBase database (v20.05) and the SwissProt UniprotKB database retrieved on January 26^th^, 2017 [45]. Repeat library sequences were removed as non-TE host genes if their best hit to a SwissProt sequence had a higher score than their best hit to a RepBase sequence. The remaining sequences were then assigned to a TE taxon when possible. A repeat library sequence was assigned to the same taxon as a REPET-formatted RepBase nucleotide or protein sequence if it had an alignment (BLASTN or BLASTX) covering 80% of the sequence for a nucleotide alignment, or had an evalue less than 1e^-10^ for a protein alignment.

The PASTEClassifier.py tool (PASTEC) from REPET version 2.2 [46] provided further information for repeat classification. The annotation information, for all sequences flagged as potentially chimeric by PASTEC, was manually reviewed. Where real chimeric sequences were verified (formed from the fusion of TEs from multiple taxa), the sequences were annotated as unknown. Otherwise, they were classified based on the methodology of [44]. Any sequences flagged as ‘rDNA’ by PASTEC were removed from the final library. Sequences categorized as Miniature Inverted-Repeat Transposable Elements were classified as Class II (DNA) elements. In a penultimate step, dotplots of all sequences were reviewed using the Geneious software package [47], and any repeats showing evidence of being composed predominantly of satellite repeat motifs were classified as such. Finally, classification information was removed from any sequences shorter than 80 bp, as suggested by [44]. The final Chinook repeat library contained 2,419 sequences, of which 1,165 (48%) were classified.

The repetitive sequence library was used to mask the genome using RepeatMasker version 4.0.7 [48], RMBlast version 2.2.28+, and Tandem Repeats Finder 4.09 [49]. The following parameters were used with RepeatMasker: -gff, -x, and -excln. The composition of repetitive elements in the genome was then extracted and compiled from the output from RepeatMasker. Circos [50] was used to plot the chromosomes, homeologous blocks, centromere positions, genetic map, average percent identity (million bp windows), and the fraction of repetitive nucleotides in million bp windows.

### Gene content

To assess the completeness of the Chinook salmon genome, a benchmarking universal single-copy orthologs (BUSCO) analysis was performed using BUSCO version 3 [51]. The actinopterygii_odb9 database was used in this analysis. The following parameters were used: -m geno, and -sp zebrafish.

To estimate the number of genes in the genome, a transcriptome was generated from eighteen RNA-seq libraries (see above for tissues used). First, STAR version 2.5.1b [52] was used to align the RNA-seq libraries to the repeat-masked genome (chromosomes only, and a version where repeats were masked with X’s) using the following parameters:—runMode alignReads,—outSAMstrandField intronMotif,—outFilterIntronMotifs RemoveNoncanonical, and—outSAMtype BAM Unsorted SortedByCoordinate.

After the reads were aligned to the genome, transcripts were identified with Cufflinks version 2.21 [53]. Individual annotations were created with Cufflinks using the -u and—total-hits-norm flags. This step produced individual annotations files that were merged with the Cuffmerge command. A script (cufflinks_gtf_genome_to_cdna_fasta.pl) from TransDecoder version 5.0.1 [54] was used to convert the merged annotation file to a sequence file with all of the transcripts, and another script (cufflinks_gtf_to alignment_gff3.pl) was used to convert the annotations into another annotation format.

Potential open reading frames (ORFs) were identified from the transcripts using the TransDecoder utility LongOrfs (with parameter -m 30). The longest peptide ORFs that were generated from the LongOrfs utility, were aligned to the UniProt database using BLASTP (-max_target_seqs 1, -oufmt 6, -evalue 1e-5). The ORFs were also aligned to the PfamA database [55] using hmmscan [56] in order to detect remote homology. The outputs from these alignments were then input into the Predict utility of TransDecoder to remove transcripts without evidence of protein homology or ORFs.

Homology information was also used to identify gene loci from the filtered transcripts. For each loci, only the best ORF, based on size, was retained. In turn, the subset of transcripts were then filtered based on keywords (e.g. transposon, long terminal, repeat, gag, bpol, long interspersed element, etc.) from their annotation to remove transposable elements. Putative splice-variants were also removed, with only the longest retained.

## Results and discussion

Assuming that the Chinook salmon genome size is 2.4 billion bp (as estimated by the count of all nucleotides in the final assembly), the total sequencing coverage from all technologies was around 202x before quality control. Table 2, describes the contributions from each of the different sequencing libraries. Roughly 73x coverage from two paired-end libraries and three mate-pair libraries was used to generate the initial genome assembly with the ALLPATHS-LG program. The contig N50 for the initial assembly was 14.6 kb, and the scaffold N50 was 1.086 Mb.

**Table 2:** Sequencing libraries.

Library	Number of Sequences (both directions)	Coverage	Length	Sequencing Technology
Paired-End1	362,760,616	37.79x	250	HiSeq Rapid
Paired-End2	366,140,468	38.90x	250	HiSeq Rapid
Mate-pair 3Kb	554,493,924	28.88x	125	HiSeq 2500
Mate-pair 5Kb	612,051,930	31.88x	125	HiSeq 2500
Mate-pair 10Kb	580,210,620	30.22x	125	HiSeq 2500
PacBio	-	34.89x	-	PacBio

After adding the PacBio data using the PBJelly software, the contig N50 increased to 149.7 kb and the scaffold N50 increased to 1.138 Mb. After an additional incorporation of the same PacBio data using SSPACE, the contig N50 increased again to 165.6 kb and the scaffold size increased to 2.192 Mb. These scaffolds were then ordered using two genetic maps and two reference genomes (rainbow trout and Atlantic salmon). The ordering and orientation of a scaffold was determined by synteny between the two reference genomes and the genetic map position(s) of markers that were aligned to the scaffolds. Approximately 73 percent of the assembled genome was placed onto 34 chromosomes in this manner, and was submitted to the National Center for Biotechnology Information (BioProject accession: PRJNA416144, Genome assembly accession: GCA_002872995.1). The rest of the scaffolds were included as part of the submission, but as unplaced scaffolds.

To assess the completeness of the genome, an analysis (BUSCO) was performed to identify the number of genes that are missing from the genome. The BUSCO analysis revealed that 90.3% of 4584 Actinopterygii genes, used to interrogate the completeness of the genome assembly, were found as complete genes in the Chinook salmon scaffolds. There were 2.1% fragmented and 7.6% missing genes. After placing the scaffolds onto chromosomes, 84.9% of the genes were still found to be complete on the chromosomes (1.7% fragmented, 13.4% missing).

A likely source of the missing genes is scaffold fragmentation, where highly similar regions of the genome interfere with scaffold generation. These sections of the genome contain enough differences to distinguish that there are two genomic regions, but enough similarity to make placing sequences to one scaffold versus the other difficult. This results in fragmented scaffolds that may be filtered based on length, or the small length may prevent the BUSCO analysis from identifying them as gene fragments. It is difficult to estimate the expected percent of the genome which might have enough sequence similarity to cause this phenomenon, as they may be underrepresented in genome assemblies.

During transcriptome analysis, a final set of 36,216 gene transcripts were identified from 18 tissues. The transcript dataset was generated from the same individual that was used to generate the reference genome, and consequently does not contain allelic variants because the individual was gynogenetic. Initially, 226,556 transcripts were identified and 3,155,777 ORFs were identified in these transcripts. After filtering based on homology, 41,411 transcripts were retained. After filtering for transposable elements, 41,189 transcripts were retained. The 36,216 final transcripts were obtained after removing splice-variants. This is similar to the number of genes (37,206) that were found for the Atlantic salmon [34], but lower than the number of genes (46,585) found in the rainbow trout genome [57]. Please note that the NCBI has agreed to generate a standardized annotation of this reference genome.

**Fig 1:**
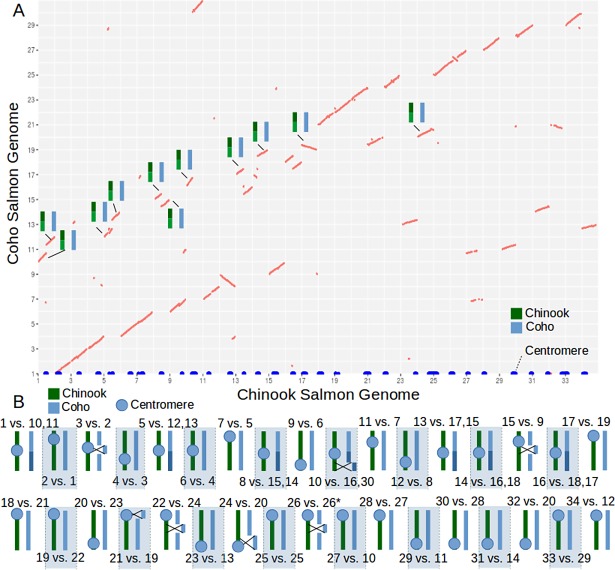
Comparison between Chinook and coho salmon genomes. A) An alignment dotplot between the Chinook and coho salmon genomes after filtering nonlinear alignments. On the x-axis the centromere locations have been plotted for each chromosome (shown in blue). Coho fusions (relative to the most common ancestor) are illustrated on the dotplot by illustrations of chromosomes. B) A diagram depicting the Chinook fusions (and one coho fission on chromosome 8). The diagram has the approximate locations of the centromere, inversions between species, and labels of the chromosomes involved. *Inversion likely due to an assembly error in the coho reference genome.

When the Chinook salmon genome sequence was aligned to the coho salmon reference genome, the genomic rearrangements and fusion events for the Chinook salmon become apparent (Fig 1). It was thought that there were 11 coho specific fusions, 1 coho specific fission, and 6 Chinook fusions relative to the most common ancestor of the Chinook and coho salmon [58]. All of these previously reported events were supported by Fig 1 and were expected since the genomes were constructed with the same genetic maps used in the previous study. There were seven major inversions identified between the Chinook and coho genomes, although the inversion on chromosome 26 (for Chinook and coho) is likely an assembly error on the coho reference genome (BF Koop, personal communication).

**Fig 2:**
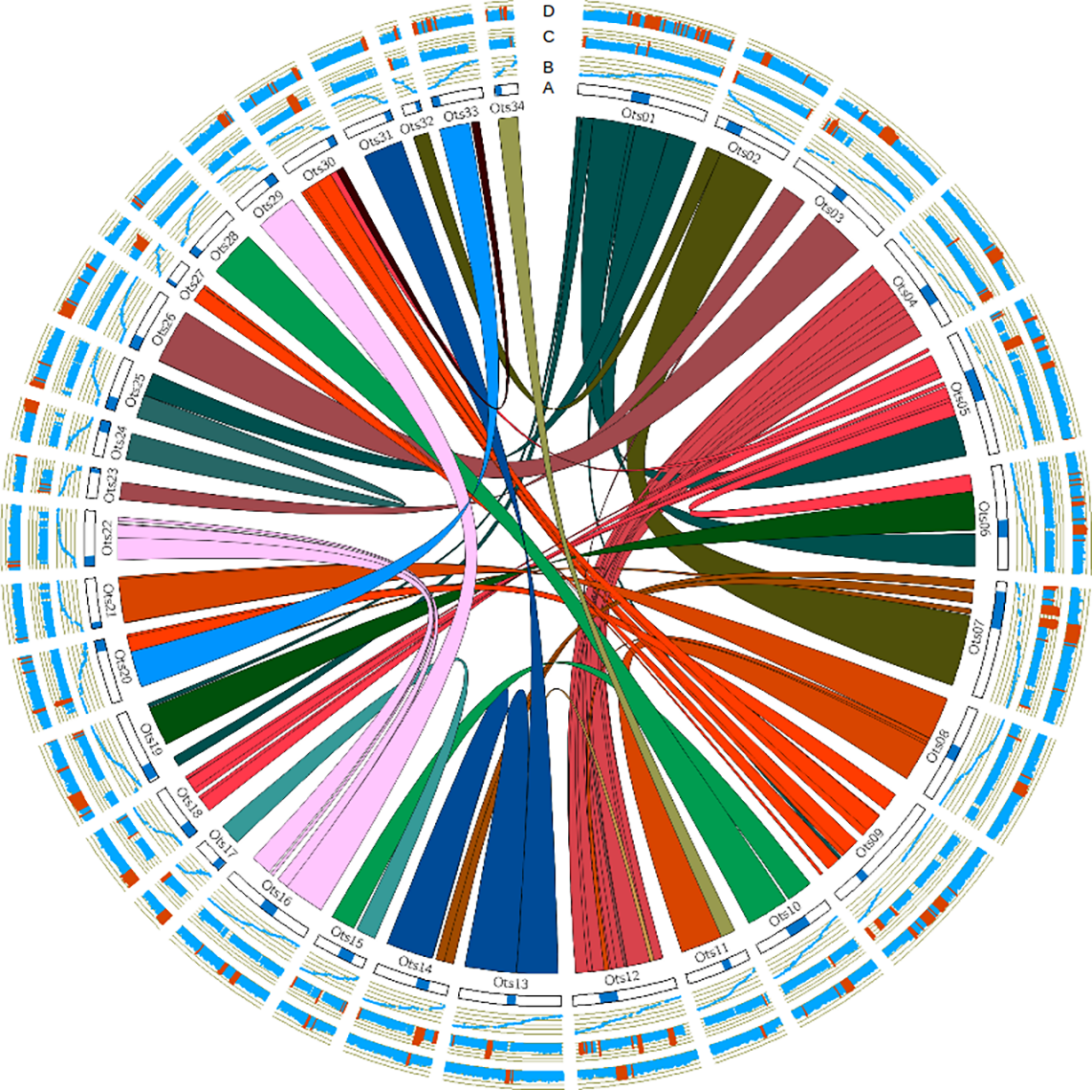
Chinook salmon circos plot. The interior links in the Circos plot, depict the duplicated (homeologous) blocks in the Chinook salmon genome defined by SyMAP. Only blocks larger than 2 million bp are shown. A) An illustration of the chromosomes with the centromere locations shown as blue filled circles (as defined by McKinney [33]). B) A scatterplot of the alignments between the McKinney [33] genetic map and the genome after filtering. The scale is in fractions of the total centimorgan length, with zero at the bottom and one at the top. C) A bar plot of the percent identity of the alignments between the blocks identified with SyMAP. The percent identity was weighted by alignment length and scored across million bp windows. The scale goes from 75 to 100 percent identity, and windows with greater than 90 percent identity were highlighted orange. D) A bar plot of the fraction of repetitive sequences found throughout the genome in million bp windows. The scale for this plot is from zero to one, with fractions of above 0.65 shown in orange.

When the Chinook genome was aligned to itself, the alignments were used by SyMAP to identify duplicated regions of the genome in blocks. SyMAP identified 170 blocks, and these blocks are highlighted in a Circos plot in Fig 2. Fig 2 also shows the centromere locations and the genetic map alignments used in assembling the genome [33]. This was shown to illustrate the close relationship with the genome sequence and the genetic map.

High similarity was often seen near the ends of the chromosomes between homeologous (duplicated) chromosomes (Fig 2), as it has been reported elsewhere for Atlantic salmon [34]. This is thought to occur because of recombination between homeologous chromosomes still occurring in salmonids [17]. These regions are likely to be incomplete because high sequence similarity between duplicated chromosomes and repetitive elements often collapse assemblies [59] and these regions are often missing in genetic maps [17].

Over 56 percent of the genome was found to be composed of repetitive elements (S1 Table), but the regions with the highest repetitive DNA were often found near centromeres (Fig 2). Repetitive sequences are thought to play a role in centromere function and they have been found and conserved extensively in eukaryotic centromere regions (but not conserved between species) [60]. A potentially ancestral centromere can be seen on Ots01, near the edge of the homeologous block between Ots01 and Ots25 (Fig 2). Ancestral centromeres are thought to lose repetitive sequence over time and likewise new centromeres are thought to gain repetitive sequences [60]. The 56 percent repetitive DNA found in the Chinook genome is slightly lower than the 58–60 percent repetitive DNA found in Atlantic salmon [34], and quite a bit higher than the 38 percent (non-TE repeats, low complexity regions, and small RNA pseudogenes) found in rainbow trout [57].

With new insights regarding the precise location of chromosomal fusions/fissions, defined duplicated blocks, and the identification of highly repetitive regions in the Chinook salmon genome, it can be appreciated that the assembled genome offers a new resource for researchers trying to understand evolutionary phenomenon. The evolutionary questions range from understanding what parts of the genome influence or underlie traits of interest in different populations or commercial lineages, to more esoteric questions regarding how chromosomes have evolved in the Salmonidae family. There are also questions that a genome sequence can help answer, that go beyond research and commercial interests.

The unravelling of biological and environmental factors affecting Chinook salmon abundance, as well as the management of the species to ensure maintenance of intraspecific diversity in the face of ongoing exploitation, will all likely be informed by research enabled by the genome sequence. For management, the identification of nucleotide variation can help define populations, and facilitates the management of mixed-stock harvest to meet conservation goals for individual populations. The genome will provide fisheries researchers and managers the full spectrum of nucleotide sequence variation to exploit in research into the adaptive capacity of the species (such as that obtained from re-sequencing data). This may be particularly important as global climate change increasingly impacts the vital coastal ecosystems upon which Chinook salmon and their prey and predator species depend.

The genome sequence will also be valuable when integrating information from other species. There are currently reference genomes in the Salmonidae family from rainbow trout, Atlantic salmon, and coho salmon in the National Center for Biotechnology Information database. There are plans to sequence the genomes for many other species in this family as well. This makes novel discoveries, found in one species more translatable between the species and facilitates making novel discoveries associated with the biology of Chinook salmon.

## Supporting information

S1 Table Repeat content in the Chinook salmon genome. Transposable element (TE) abundances were reported in the RepeatMasker output, and because individual TEs annotations in the genome may occasionally overlap, the reported values are not necessarily additive. The percent coverage is based on the the base-pair coverage and the genome excluding tracks of more than 19 consecutive unknown nucleotides (represented as N’s in the genome sequence). RepeatMasker associated 53.07% of the genome with interspersed repeats and masked 56.48% of the genome as repeat-derived.
(DOCX)

S1 File A compressed folder containing all of the perl and python scripts used in this study. To view the contents of this folder, please unzip and untar the file. The readme.txt file has a comprehensive description of how to use these scripts and finer detail on the methodology of placing scaffolds onto chromosomes.
(TAR)

## References

[1] Healey MC. Life History of Chinook Salmon (Oncorhynchus tshawytscha). In: Groot C, Margolis L, editors. Pacific Salmon Life Histories. 1998th ed. Vancouver, BC: UBC Press; 1998 p. 311–95.

[2] Willson MF, Halupka KC. Anadromous Fish as Keystone Species in Vertebrate Communities. Conserv Biol. 1995;9(3):489–97.

[3] Behnke R. Chinook Salmon Oncorhynchus tshawytscha In: Trout and Salmon of North America. Free Press; 2002 p. 25–31.

[4] US EPA R 10. Chinook Salmon [Internet]. US EPA. 2013 [cited 2018 Feb 28]. Available from: https://www.epa.gov/salish-sea/chinook-salmon

[5] Irvine JR, Fukuwaka M. Pacific salmon abundance trends and climate change. ICES J Mar Sci. 2011 7 1;68(6):1122–30.

[6] Washington Department of Fish & Wildlife [Internet]. 2017 [cited 2017 Dec 19]. Available from: http://wdfw.wa.gov/

[7] Columbia Basin Research [Internet]. Welcome to Columbia Basin Research | Columbia Basin Research. 2017 [cited 2017 Dec 19]. Available from: http://www.cbr.washington.edu/

[8] Qin Y, Kaeriyama M. Feeding Habits and Trophic Levels of Pacific Salmon (Oncorhynchus spp.) in the North Pacific Ocean. North Pac Anadromous Fish Commision. 2016;Bulletin 6:469–81.

[9] Bond NA, Cronin MF, Freeland H, Mantua N. Causes and impacts of the 2014 warm anomaly in the NE Pacific. Geophys Res Lett. 2015 5 16;42(9):2015GL063306.

[10] Chasco BE, Kaplan IC, Thomas AC, Acevedo-Gutiérrez A, Noren DP, Ford MJ, et al Competing tradeoffs between increasing marine mammal predation and fisheries harvest of Chinook salmon. Sci Rep. 2017 11 20;7(1):15439 doi: 10.1038/s41598-017-14984-8 29158502 10.1038/s41598-017-14984-8 PMC5696463

[11] Heard WR, Shevlyakov E, Zikunova OV, McNicol RE. Chinook salmon—trends in abundance and biological characteristics. North Pac Anadromous Fish Commision. 2007;Bulletin 4:77–91.

[12] Ciancio JE, Rossi CR, Pascual M, Anderson E, Garza JC. The invasion of an Atlantic Ocean river basin in Patagonia by Chinook salmon: new insights from SNPs. Biol Invasions. 2015 10 1;17(10):2989–98.

[13] Hecht BC, Matala AP, Hess JE, Narum SR. Environmental adaptation in Chinook salmon (Oncorhynchus tshawytscha) throughout their North American range. Mol Ecol. 2015 11;24(22):5573–95. doi: 10.1111/mec.13409 26465117 10.1111/mec.13409

[14] Mantua NJ, Crozier LG, Reed TE, Schindler DE, Waples RS. Response of chinook salmon to climate change. Nat Clim Change. 2015 7;5(7):613.

[15] Allendorf FW, Thorgaard GH. Tetraploidy and the Evolution of Salmonid Fishes In: Turner BJ, editor. Evolutionary Genetics of Fishes [Internet]. Springer US; 1984 [cited 2015 Mar 17]. p. 1–53. (Monographs in Evolutionary Biology). Available from: http://link.springer.com/chapter/10.1007/978-1-4684-4652-4_1

[16] Macqueen DJ, Johnston IA. A well-constrained estimate for the timing of the salmonid whole genome duplication reveals major decoupling from species diversification. Proc R Soc B Biol Sci [Internet]. 2014 3 7 [cited 2015 Mar 17];281(1778). Available from: http://www.ncbi.nlm.nih.gov/pmc/articles/PMC3906940/10.1098/rspb.2013.2881 PMC3906940 24452024

[17] Allendorf FW, Bassham S, Cresko WA, Limborg MT, Seeb LW, Seeb JE. Effects of Crossovers Between Homeologs on Inheritance and Population Genomics in Polyploid-Derived Salmonid Fishes. J Hered. 2015 5 1;106(3):217–27. doi: 10.1093/jhered/esv015 25838153 10.1093/jhered/esv015

[18] Angeloni F, Wagemaker N, Vergeer P, Ouborg J. Genomic toolboxes for conservation biologists. Evol Appl. 2012 2;5(2):130–43. doi: 10.1111/j.1752-4571.2011.00217.x 25568036 10.1111/j.1752-4571.2011.00217.x PMC3353346

[19] Khan S, Nabi G, Ullah MW, Yousaf M, Manan S, Siddique R, et al Overview on the Role of Advance Genomics in Conservation Biology of Endangered Species [Internet]. International Journal of Genomics. 2016 [cited 2018 Mar 2]. Available from: https://www.hindawi.com/journals/ijg/2016/3460416/10.1155/2016/3460416 PMC5153469 28025636

[20] Gutierrez AP, Yáñez JM, Fukui S, Swift B, Davidson WS. Genome-Wide Association Study (GWAS) for Growth Rate and Age at Sexual Maturation in Atlantic Salmon (Salmo salar). PLoS ONE [Internet]. 2015 3 10 [cited 2018 Mar 2];10(3). Available from: https://www.ncbi.nlm.nih.gov/pmc/articles/PMC4355585/10.1371/journal.pone.0119730 PMC4355585 25757012

[21] Yue GH. Recent advances of genome mapping and marker-assisted selection in aquaculture. Fish Fish. 2014 9 1;15(3):376–96.

[22] Barría A, Christensen KA, Yoshida GM, Correa K, Jedlicki A, Lhorente JP, et al Genomic Predictions and Genome-Wide Association Study of Resistance Against Piscirickettsia salmonis in Coho Salmon (Oncorhynchus kisutch) Using ddRAD Sequencing. G3 Genes Genomes Genet. 2018 2 13;g3.200053.2018. 10.1534/g3.118.200053 PMC5873909 29440129

[23] Zhang H, Tan E, Suzuki Y, Hirose Y, Kinoshita S, Okano H, et al Dramatic improvement in genome assembly achieved using doubled-haploid genomes. Sci Rep. 2014 10 27;4:6780 doi: 10.1038/srep06780 25345569 10.1038/srep06780 PMC5381364

[24] Quillet E, Garcia P, Guyomard R. Analysis of the production of all homozygous lines of rainbow trout by gynogenesis. J Exp Zool. 1991 3 1;257(3):367–74.

[25] Andrews S. FastQC [Internet]. Babraham Bioinformatics—FastQC A Quality Control tool for High Throughput Sequence Data. 2016 [cited 2017 Dec 19]. Available from: https://www.bioinformatics.babraham.ac.uk/projects/fastqc/

[26] Bolger AM, Lohse M, Usadel B. Trimmomatic: a flexible trimmer for Illumina sequence data. Bioinformatics. 2014 8 1;30(15):2114–20. doi: 10.1093/bioinformatics/btu170 24695404 10.1093/bioinformatics/btu170 PMC4103590

[27] Gnerre S, MacCallum I, Przybylski D, Ribeiro FJ, Burton JN, Walker BJ, et al High-quality draft assemblies of mammalian genomes from massively parallel sequence data. Proc Natl Acad Sci. 2011 1 25;108(4):1513–8. doi: 10.1073/pnas.1017351108 21187386 10.1073/pnas.1017351108 PMC3029755

[28] English AC, Richards S, Han Y, Wang M, Vee V, Qu J, et al Mind the Gap: Upgrading Genomes with Pacific Biosciences RS Long-Read Sequencing Technology. PLOS ONE. 2012 11 21;7(11):e47768 doi: 10.1371/journal.pone.0047768 23185243 10.1371/journal.pone.0047768 PMC3504050

[29] blasr: BLASR: The PacBio® long read aligner [Internet]. Pacific Biosciences; 2017 [cited 2017 Dec 19]. Available from: https://github.com/PacificBiosciences/blasr

[30] Koren S, Walenz BP, Berlin K, Miller JR, Bergman NH, Phillippy AM. Canu: scalable and accurate long-read assembly via adaptive k-mer weighting and repeat separation. Genome Res. 2017 5 1;27(5):722–36. doi: 10.1101/gr.215087.116 28298431 10.1101/gr.215087.116 PMC5411767

[31] Boetzer M, Henkel CV, Jansen HJ, Butler D, Pirovano W. Scaffolding pre-assembled contigs using SSPACE. Bioinformatics. 2011 2 15;27(4):578–9. doi: 10.1093/bioinformatics/btq683 21149342 10.1093/bioinformatics/btq683

[32] Brieuc MSO, Waters CD, Seeb JE, Naish KA. A dense linkage map for Chinook salmon (Oncorhynchus tshawytscha) reveals variable chromosomal divergence after an ancestral whole genome duplication event. G3 Bethesda Md. 2014 3 20;4(3):447–60. 10.1534/g3.113.009316 PMC3962484 24381192

[33] McKinney GJ, Seeb LW, Larson WA, Gomez-Uchida D, Limborg MT, Brieuc MSO, et al An integrated linkage map reveals candidate genes underlying adaptive variation in Chinook salmon (Oncorhynchus tshawytscha). Mol Ecol Resour. 2016 5;16(3):769–83. doi: 10.1111/1755-0998.12479 26490135 10.1111/1755-0998.12479

[34] Lien S, Koop BF, Sandve SR, Miller JR, Kent MP, Nome T, et al The Atlantic salmon genome provides insights into rediploidization. Nature. 2016 5 12;533(7602):200–5. doi: 10.1038/nature17164 27088604 10.1038/nature17164 PMC8127823

[35] Li H. Aligning sequence reads, clone sequences and assembly contigs with BWA-MEM. ArXiv13033997 Q-Bio [Internet]. 2013 Mar 16 [cited 2017 Dec 19]; Available from: http://arxiv.org/abs/1303.3997

[36] Camacho C, Coulouris G, Avagyan V, Ma N, Papadopoulos J, Bealer K, et al BLAST+: architecture and applications. BMC Bioinformatics. 2009 12 15;10(1):1–9. 20003500 10.1186/1471-2105-10-421 PMC2803857

[37] Morgulis A, Coulouris G, Raytselis Y, Madden TL, Agarwala R, Schäffer AA. Database indexing for production MegaBLAST searches. Bioinforma Oxf Engl. 2008 8 15;24(16):1757–64. 10.1093/bioinformatics/btn322 PMC2696921 18567917

[38] Kurtz S, Phillippy A, Delcher AL, Smoot M, Shumway M, Antonescu C, et al Versatile and open software for comparing large genomes. Genome Biol. 2004;5(2):R12 doi: 10.1186/gb-2004-5-2-r12 14759262 10.1186/gb-2004-5-2-r12 PMC395750

[39] R Core Team. R: A Language and Environment for Statistical Computing [Internet]. R Foundation for Statistical Computing; 2017 Available from: https://www.R-project.org

[40] Wickham H. ggplot2: Elegant Graphics for Data Analysis. 1st ed. 2009. Corr. 3rd printing 2010 edition. New York: Springer; 2010. 213 p.

[41] Soderlund C, Bomhoff M, Nelson WM. SyMAP v3.4: a turnkey synteny system with application to plant genomes. Nucleic Acids Res. 2011 5;39(10):e68 doi: 10.1093/nar/gkr123 21398631 10.1093/nar/gkr123 PMC3105427

[42] Jurka J, Kapitonov VV, Pavlicek A, Klonowski P, Kohany O, Walichiewicz J. Repbase Update, a database of eukaryotic repetitive elements. Cytogenet Genome Res. 2005;110(1–4):462–7. doi: 10.1159/000084979 16093699 10.1159/000084979

[43] Smit A, Hubley R. RepeatModeler Open-1.0 [Internet]. 2013 [cited 2017 Dec 18]. Available from: http://www.repeatmasker.org/

[44] Wicker T, Sabot F, Hua-Van A, Bennetzen JL, Capy P, Chalhoub B, et al A unified classification system for eukaryotic transposable elements. Nat Rev Genet. 2007 12;8(12):973–82. doi: 10.1038/nrg2165 17984973 10.1038/nrg2165

[45] The UniProt Consortium. UniProt: the universal protein knowledgebase. Nucleic Acids Res. 2017 1 4;45(D1):D158–69. doi: 10.1093/nar/gkw1099 27899622 10.1093/nar/gkw1099 PMC5210571

[46] Flutre T, Duprat E, Feuillet C, Quesneville H. Considering Transposable Element Diversification in De Novo Annotation Approaches. PLOS ONE. 2011 1 31;6(1):e16526 doi: 10.1371/journal.pone.0016526 21304975 10.1371/journal.pone.0016526 PMC3031573

[47] Kearse M, Moir R, Wilson A, Stones-Havas S, Cheung M, Sturrock S, et al Geneious Basic: an integrated and extendable desktop software platform for the organization and analysis of sequence data. Bioinforma Oxf Engl. 2012 6 15;28(12):1647–9. 10.1093/bioinformatics/bts199 PMC3371832 22543367

[48] Smit A, Hubley R, Green P. RepeatMasker Open-4.0 [Internet]. 2013 [cited 2017 Dec 18]. Available from: http://www.repeatmasker.org/

[49] Benson G. Tandem repeats finder: a program to analyze DNA sequences. Nucleic Acids Res. 1999 1 15;27(2):573–80. 9862982 10.1093/nar/27.2.573 PMC148217

[50] Krzywinski MI, Schein JE, Birol I, Connors J, Gascoyne R, Horsman D, et al Circos: An information aesthetic for comparative genomics. Genome Res [Internet]. 2009 6 18 [cited 2015 May 21]; Available from: http://genome.cshlp.org/content/early/2009/06/15/gr.092759.109 10.1101/gr.092759.109 PMC2752132 19541911

[51] Simão FA, Waterhouse RM, Ioannidis P, Kriventseva EV, Zdobnov EM. BUSCO: assessing genome assembly and annotation completeness with single-copy orthologs. Bioinformatics. 2015 10 1;31(19):3210–2. doi: 10.1093/bioinformatics/btv351 26059717 10.1093/bioinformatics/btv351

[52] Dobin A, Davis CA, Schlesinger F, Drenkow J, Zaleski C, Jha S, et al STAR: ultrafast universal RNA-seq aligner. Bioinformatics. 2013 1;29(1):15–21. doi: 10.1093/bioinformatics/bts635 23104886 10.1093/bioinformatics/bts635 PMC3530905

[53] Trapnell C, Williams BA, Pertea G, Mortazavi A, Kwan G, van Baren MJ, et al Transcript assembly and abundance estimation from RNA-Seq reveals thousands of new transcripts and switching among isoforms. Nat Biotechnol. 2010 5;28(5):511–5. doi: 10.1038/nbt.1621 20436464 10.1038/nbt.1621 PMC3146043

[54] Haas BJ, Papanicolaou A, Yassour M, Grabherr M, Blood PD, Bowden J, et al De novo transcript sequence reconstruction from RNA-Seq: reference generation and analysis with Trinity. Nat Protoc. 2013 8;8(8): doi: 10.1038/nprot.2013.084 23845962 10.1038/nprot.2013.084 PMC3875132

[55] Finn RD, Tate J, Mistry J, Coggill PC, Sammut SJ, Hotz H-R, et al The Pfam protein families database. Nucleic Acids Res. 2008 1;36(Database issue):D281–8. doi: 10.1093/nar/gkm960 18039703 10.1093/nar/gkm960 PMC2238907

[56] HMMER [Internet]. 2017 [cited 2017 Dec 19]. Available from: http://hmmer.org/

[57] Berthelot C, Brunet F, Chalopin D, Juanchich A, Bernard M, Noël B, et al The rainbow trout genome provides novel insights into evolution after whole-genome duplication in vertebrates. Nat Commun [Internet]. 2014 4 22 [cited 2015 Mar 18];5 Available from: http://www.nature.com/ncomms/2014/140422/ncomms4657/full/ncomms4657.html 10.1038/ncomms4657 PMC4071752 24755649

[58] Sutherland BJG, Gosselin T, Normandeau E, Lamothe M, Isabel N, Audet C, et al Salmonid Chromosome Evolution as Revealed by a Novel Method for Comparing RADseq Linkage Maps. Genome Biol Evol. 2016 12 1;8(12):3600–17. doi: 10.1093/gbe/evw262 28173098 10.1093/gbe/evw262 PMC5381510

[59] Salzberg SL, Yorke JA. Beware of mis-assembled genomes. Bioinforma Oxf Engl. 2005 12 15;21(24):4320–1. 10.1093/bioinformatics/bti769 16332717

[60] McKinley KL, Cheeseman IM. The molecular basis for centromere identity and function. Nat Rev Mol Cell Biol. 2016 1;17(1):16–29. doi: 10.1038/nrm.2015.5 26601620 10.1038/nrm.2015.5 PMC8603311

