# Supplementary material for: Chinook salmon (Oncorhynchus tshawytscha) genome and transcriptome
Source: PLoS One. 2018 Apr 5;13(4):e0195461. doi: 10.1371/journal.pone.0195461 (PMC5886536; doi:10.1371/journal.pone.0195461)
Supplement: S1 Table — Transposable element (TE) abundances were reported in the RepeatMasker output, and because individual TEs annotations in the genome may occasionally overlap, the reported values are not necessarily additive. The percent coverage is based on the the base-pair coverage and the genome excluding tracks of more than 19 consecutive unknown nucleotides (represented as N’s in the genome sequence). RepeatMasker associated 53.07% of the genome with interspersed repeats and masked 56.48% of the genome as repeat-derived. (DOCX) [file pone.0195461.s001.docx]

| **Repeat Type** | **Order** | **Superfamily** | **Coverage (Mbp)** | **Coverage (%)** |
| --- | --- | --- | --- | --- |
| **Class I TEs** | **All** | **All** | **304.57** | **17.48** |
|  | **LTR** | **All** | **44.54** | **2.56** |
|  |  | Gypsy | 32.67 | 1.87 |
|  |  | ERV | 6.60 | 0.38 |
|  |  | Copia | 3.69 | 0.21 |
|  |  | Bel-Pao | 1.58 | 0.09 |
|  | **DIRS** | **DIRS** | **2.05** | **0.12** |
|  | **PLE** | **Penelope** | **2.74** | **0.16** |
|  | **LINE** | **All** | **250.77** | **14.39** |
|  |  | Rex1 | 98.28 | 5.64 |
|  |  | Crack | 82.98 | 4.76 |
|  |  | L2 | 25.54 | 1.47 |
|  |  | RTEX | 14.65 | 0.84 |
|  |  | Tx1 | 12.96 | 0.74 |
|  |  | L1 | 9.84 | 0.56 |
|  |  | Nimb | 3.77 | 0.22 |
|  |  | Hero | 0.46 | 0.03 |
|  |  | Keno | 1.39 | 0.08 |
|  |  | CR1 | 0.53 | 0.03 |
|  |  | RTE | 0.17 | 0.01 |
|  |  | R2 | 0.02 | 0.00 |
|  | **SINE** | **All** | **4.46** | **0.26** |
|  |  | tRNA | 2.70 | 0.15 |
|  |  | Deu | 1.76 | 0.10 |
| **Class II TEs** | **All** | **All** | **373.21** | **21.42** |
|  | **TIR** | **All** | **318.25** | **18.26** |
|  |  | Tc1-Mariner | 262.05 | 15.04 |
|  |  | hAT | 41.70 | 2.39 |
|  |  | PiggyBac | 6.00 | 0.34 |
|  |  | CMC-EnSpm | 3.12 | 0.18 |
|  |  | Harbinger | 1.49 | 0.09 |
|  |  | IS3EU | 1.21 | 0.07 |
|  |  | Ginger | 0.88 | 0.05 |
|  |  | Kolobok | 0.74 | 0.04 |
|  |  | Sola | 0.86 | 0.05 |
|  |  | ISL2EU | 0.21 | 0.01 |
|  | **Dada** | **Dada** | **0.03** | **0.00** |
|  | **Crypton** | **Crypton** | **2.91** | **0.17** |
|  | **Maverick** | **Maverick** | **1.53** | **0.09** |
| **Unclassified** |  |  | **278.19** | **15.96** |
| **Satellites** |  |  | **11.08** | **0.64** |
| **Simple Repeats** |  |  | **39.87** | **2.29** |
| **Low complexity** |  |  | **10.36** | **0.59** |
